# Newly formed cystic lesions for the development of pneumomediastinum in Pneumocystis jirovecii pneumonia

**DOI:** 10.1186/1471-2334-9-171

**Published:** 2009-10-18

**Authors:** Ju-Yeon Cho, Dong-Min Kim, Yong Eun Kwon, Sung Ho Yoon, Seung Il Lee

**Affiliations:** 1 Department of Internal Medicine1, Chosun University, College of Medicine, Republic of Korea

## Abstract

**Background:**

*Pneumocystis jirovecii*, formerly named *Pneumocystis carinii*, is one of the most common opportunistic infections in human immunodeficiency virus (HIV)-infected patients.

**Case presentations:**

We encountered two cases of spontaneous pneumomediastinum with subcutaneous emphysema in HIV-infected patients being treated for *Pneumocystis jirovecii *pneumonia with trimethoprim/sulfamethoxazole.

**Conclusion:**

Clinicians should be aware that cystic lesions and bronchiectasis can develop in spite of trimethoprim/sulfamethoxazole treatment for *P. jirovecii *pneumonia. The newly formed bronchiectasis and cyst formation that were noted in follow up high resolution computed tomography (HRCT) but were not visible on HRCT at admission could be risk factors for the development of pneumothorax or pneumomediastinum with subcutaneous emphysema in HIV-patients.

## Background

*Pneumocystis jirovecii*, formerly named *Pneumocystis carinii*, is one of the most common opportunistic infections in human immunodeficiency virus (HIV)-infected patients [1,2]. Spontaneous pneumothorax has been recognized as a frequent complication in patients with *P. jirovecii *pneumonia (PCP) since it was first described in 1984 [3], and pneumomediastinum is an uncommon complication associated with pneumothorax in the aforementioned population. We report two cases of spontaneous pneumomediastinum with subcutaneous emphysema in HIV-infected patients being treated for *P. jirovecii *pneumonia with trimethoprim/sulfamethoxazole.

## Case presentation

### Case 1

A 33-year-old man presented with fever, dyspnea, and odynophagia. Five months prior to admission, the patient had been treated for dental caries at a local hospital, and at that time examination revealed seropositivity for human immunodeficiency virus. On admission, temperature was 39.0'C, pulse 92 beats per minute, respiratory rate 20 breaths per minute and blood pressure 130/80 mmHg. Physical examination revealed oral thrush, consistent with findings of extensive esophageal candidiasis in endoscopic gastroduodenscopy performed five days before admission. Laboratory data on admission revealed a WBC count of 1,760/uL, Hb 10.9 g/dL, and platelet count of 297,000/uL. Arterial blood gas analysis while breathing room air revealed PaO_2 _of 48.0 mmHg, PaCO_2 _of 32.7 mmHg, and saturation of 88.5%, and the calculated (A-a)DO_2 _was 53.7. CD4 count and HIV viral load were 4/uL and 130,000 IU/mL, respectively. Diffuse bilateral infiltrates of both lung fields were noted, and no cystic lesions were observed on the chest X-ray and high resolution computed tomography (HRCT) taken on admission. Bronchoscopic alveolar lavage for diagnosis of *P. jirovecii *was carried out, and microscopic examination of the bronchoalveolar lavage fluid obtained showed *P. jirovecii*; no other microorganisms were detected by culture. Treatment with trimethoprim/sulfamethoxazole, fluconazole and corticosteroids at standard dosages was started. The patient had never been on HAART therapy prior to admission. HAART therapy was added to the treatment on the 8^th ^hospital day. During the treatment with trimethoprim/sulfamethoxazole, pancytopenia worsened. Bone marrow biopsy revealed inflamed marrow and partial necrosis. Granulocyte colony stimulating factor was used without avail. On the 22^nd ^hospital day, the chest X-ray obtained as the patient's hypoxemia worsened revealed pneumomediastinum. HRCT showed newly formed cystic lesions in both lung fields. Pneumomediastinum was treated conservatively with high oxygen supply. CD4 cell count and HIV levels were not followed during treatment. However, as the general condition of the patient deteriorated, the patient was started on intravenous pentamidine on the 23^rd ^hospital day. On the 25^th ^hospital day, his oxygen requirement increased. Without intubation, as the patient and guardian refused the patient being put on a ventilator due to multiple economical and sociological reasons, the patient died on the 26^th ^hospital day.

### Case 2

**Figure 1:**
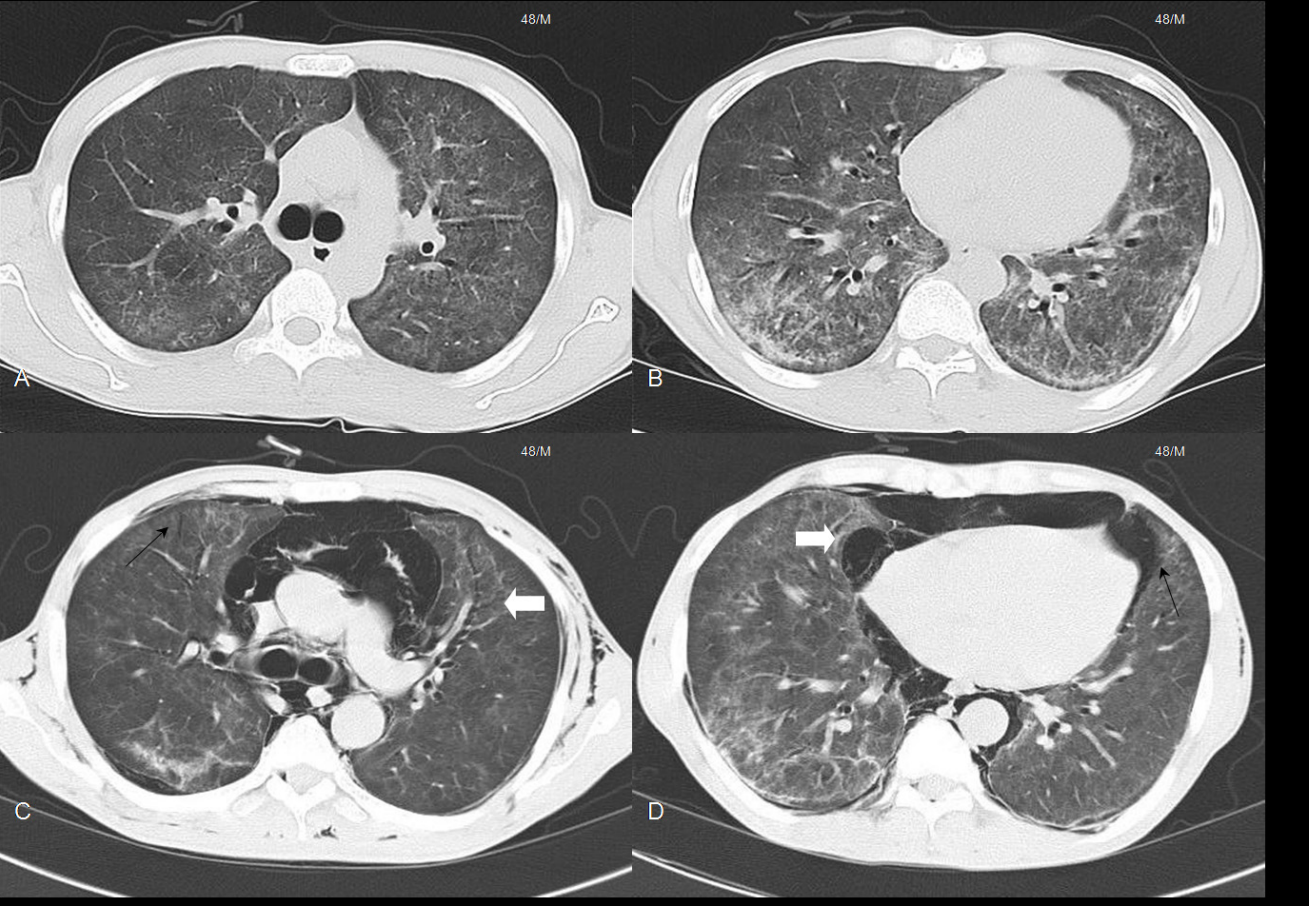
**Radiological findings of an 48-year-old man with *Pneumocystis jirovecii *pneumonia on admission and at follow up**. A. Computed tomography on admission reveals diffuse bilateral infiltrates. B. Diffuse ground glass opacities are noted on both lung fields on admission. C. High resolution computed tomography reveals newly developed bronchiectatic changes (white arrow) and parenchymal tears (black arrow) at follow up. D. Pneumomediastinum (black arrow) and cystic changes (white arrow) are seen at follow up.

A 48-year-old man presented with insidious dyspnea that had developed over a period of 2 months. The patient had been seropositive for human immunodeficiency virus in 2005 in a routine physical examination for a job position as a sailor. He was on zidovudine and didanosine for 8 months but stopped taking these antiretroviral agents at another hospital for economic reasons. On admission, temperature was 38.2'C, pulse 96 beats per minute, respiratory rate 22 breaths per minute, and blood pressure 100/60 mmHg. Physical examination was normal except for decreased breathing sound in both lung fields. Laboratory data showed a WBC count of 11,010/uL, Hb 13.6 g/dL, a platelet count of 425,000/uL, and LDH of 1,095 U/L. Arterial blood gas analysis in room air demonstrated PaO_2 _of 54.1 mmHg, PaCO_2 _of 34.4 mmHg, and saturation of 89.9%, and the calculated (A-a)DO_2 _was 45.5. CD4 count and HIV viral load were 21/uL and 260,000 IU/mL, respectively. Chest X-ray on admission revealed diffuse ground glass opacity in both lung fields. HRCT showed interlobular septal thickening of the bronchus and bronchioles without any cyst formation (Figure 1). Microscopic examination of bronchoalveolar lavage revealed *P. jiroveci*, and *Staphylococcus aureus *was detected by culture. The patient met the criteria of respiratory failure [PaO_2 _less than 70 mmHg or (A-a)DO_2 _more than 35 mmHg] and corticosteroids were co-administered with trimethoprim/sulfamethoxazole. On hospital day 4 the patient developed sudden chest pain radiating to the shoulder and neck. Chest X-ray, electrocardiography, and arterial blood gas analysis were performed. The chest X-ray revealed air lining the cardiac border, indicating development of pneumomediastinum. HRCT revealed newly developed cystic changes, bronchiectatic change, and parenchymal tear (Figure 1). The pneumomediastinum was treated conservatively with administration of high oxygen supply without the need for invasive procedures. On hospital day 10, HAART was started with lopinavir/ritonavir, lamivudine, and zidovudine. On hospital day 13, follow up HRCT revealed more aggravated pneumomediastinum, bronchiectasis and parenchymal tear in the lingular division of the left upper lobe. Trimethoprim/sulfamethoxazole was changed to intravenous pentamidine. The patient experienced nausea, vomiting, and hypoglycemia on pentamidine, leading to a further change of antibiotics to primaquine and clindamycin. The patient's dyspnea improved and no particular complications were observed. The patient was discharged on the 42^nd ^hospital day and is being followed up in the outpatient clinic.

## Discussion

The overall incidence of *P. jirovecii *pneumonia has decreased with the use of highly active antiretroviral therapy [4]. However, approximately 85% of patients with advanced HIV infections continue to experience *P. jirovecii *pneumonia in the course of their disease when management is inadequate [5]. The most common radiographic finding in *P. jirovecii *pneumonia is the presence of diffuse, bilateral perihilar interstitial infiltrates (ground-glass opacity) in both lungs [6]. Atypical radiographic manifestations of PCP include cystic spaces and bullae, adenopathy, pleural effusions and pneumothorax [7-10]. The exact mechanism behind the development of the pulmonary cysts and *P. jirovecii *is not yet known. However, various mechanisms have been proposed including direct lung destruction by *P. jirovecii*, over-distension of the lungs caused by obstructive bronchiolitis acting as a ball-valve (inflammatory exudates in the small bronchioles), interstitial emphysema and abnormal remodeling of pulmonary architecture due to interstitial fibrosis, and release of elastase and other proteolytic enzymes [5,10-12]. A review of the literature indicates that the development of spontaneous pneumomediastinum with subcutaneous emphysema in HIV patients is rare. The pathophysiology of pneumomediastinum depends on a pressure gradient between the alveoli and the lung interstitium; this leads to alveolar rupture, and consequently air in the interstitial space flows towards the mediastinum along a pressure gradient between the lung periphery and the mediastinum [13]. Other mechanisms leading to this outcome have been attributed to gas-producing microorganisms present in the pneumomediastinum, and rupture of the mucosal barrier of the esophagus or tracheobronchial tree [14]. However, how the presence of *P. jirovecii *contributes to the development of pneumomediastinum is unknown.

Spontaneous pneumothorax occurs in as many as 35% of patients with active cystic *P. jirovecii *pneumonia [8]. Even though it is not known exactly how the disease progresses to pneumothorax or pneumomediastinum, it is important to identify associated factors and be able to predict their occurrence. A history of cigarette smoking, pentamidine aerosol treatment, and detection of pneumatoceles by chest radiography, are reported risk factors associated with spontaneous pneumothorax [15,16].

In relation to the aforementioned risk factors, a 22 pack-year history of smoking was noted in one of our two patients. Pneumatoceles or cysts were not seen on the chest X-rays or HRCT scans taken on admission in either patient. However, cystic lesions and bronchiectasis developed de novo in spite of the standard trimethoprim/sulfamethoxazole treatment, and they are presumed to have developed into pneumomediastinum with subcutaneous emphysema [17]. *Staphylococcus aureus *that was cultured in the second case may have attributed to the development of pneumomediastinum [18]. However, the use of trimethoprim/sulfamethoxazole was adequate for treating *Staphylococcus aureus *without the need for an additional antibiotic [19]. Treatment of spontaneous pneumomediastinum is generally limited to observation without the need for invasive measures [20]. However, in the above patients, *P. jirovecii *pneumonia may have been an underlying cause to the development of pneumomediastinum. Therefore, the standard trimethorpim/sulfamethoxazole was analyzed as a treatment failure warranting a change of antibiotics to pentamidine.

There is no guideline regarding the treatment of the acute phase of *P. jirovecii *pneumonia in HIV-infected patients with HAART. However, administration of HAART therapy early in the acute phase of *P. jirovecii *pneumonia was done in both patients as improved survival rates in HIV-infected patients with severe *P. jirovecii *pneumonia was associated with HAART therapy [21,22]. Although the developement of pneumothorax was not anticipated in our patients when HAART therapy was initiated, Morris et al reported decreased rates of pneumothorax development in *P. jirovecii *infected HIV-patients receiving HAART therapy[21]. The possible contribution of antiretroviral therapy to the clinical worsening of the patients was considered. Wislez et al reported of acute respiratory failure following HAART in *P. jirovecii *pneumonia due to immune reconstitution inflammatory syndrome [23]. HRCT of both patients in this study did not reveal any findings relevant to the development of acute respiratory failure.

The occurrence of newly formed cystic lesions or bronchiectasis despite treatment may be risk factors for the development of pneumothorax or pneumomediastinum with subcutaneous emphysema in HIV-patients. Therefore close follow up with HRCT in HIV-patients with *P. jirovecii *pneumonia might assist in predicting the development of pneumothorax or pneumomediastinum. Our findings suggest that clinicians should be aware of the clinical importance of newly formed cystic lesions and bronchiectasis for the development of pneumomediastinum and pneumothorax in *P. jirovecii *pneumonia.

## Conclusion

In conclusion, clinicians should be aware that cystic lesions and bronchiectasis can develop in spite of trimethoprim/sulfamethoxazole treatment for *P. jirovecii *pneumonia. Newly formed bronchiectasis and cyst formation may be risk factors for the development of pneumomediastinum with subcutaneous emphysema.

## Competing interests

The authors declare that they have no competing interests.

## Authors' contributions

Ju-Yeon Cho took care of the patient in the ICU and drew

up the first draft of the report, Yong Eun Kwon, Sung Ho Yoon, and Seung Il Lee, consultant pulmonologists, made a substantial contribution to draft the manuscript

and revised the draft all over the course of submission, Dong-Min Kim conceived

of the study, participated in its design and coordination and drafted the manuscript. All authors read and approved the final manuscript.

## Pre-publication history

The pre-publication history for this paper can be accessed here:

http://www.biomedcentral.com/1471-2334/9/171/prepub
